# Acquired Activated Protein C Resistance, Thrombophilia and Adverse Pregnancy Outcomes: A Study Performed in an Irish Cohort of Pregnant Women

**DOI:** 10.1155/2011/232840

**Published:** 2011-08-14

**Authors:** Sara Sedano-Balbás, Mark Lyons, Brendan Cleary, Margaret Murray, Geraldine Gaffney, Majella Maher

**Affiliations:** ^1^Molecular Diagnostics Research Group, National Centre for Biomedical Engineering Science, National University of Ireland, Galway, Ireland; ^2^Department of Haematology, University College Hospital, Galway, Ireland; ^3^Department of Obstetrics & Gynaecology, University College Hospital, Galway, Ireland

## Abstract

The combination of thrombophilia and pregnancy increases the risk of thrombosis and the potential for adverse outcomes during pregnancy. The most significant common inherited risk factor for thrombophilia is activated protein C resistance (APCR), a poor anticoagulant response of APC in haemostasis, which is mainly caused by an inherited single-nucleotide polymorphism (SNP), factor V G1691A (FV Leiden) (FVL), referred as inherited APCR. Changes in the levels of coagulation factors: FV, FVIII, and FIX, and anticoagulant factors: protein S (PS) and protein C (PC) can alter APC function causing acquired APCR. Prothrombin G20210A and methylenetetrahydrofolate reductase (MTHFR) C677T are prothrombotic SNPs which in association with APCR can also increase the risk of thrombosis amongst Caucasians. In this study, a correlation between an acquired APCR phenotype and increased levels of factors V, VIII, and IX was demonstrated. Thrombophilic mutations amongst our acquired APCR pregnant women cohort are relatively common but do not appear to exert a severe undue adverse effect on pregnancy.

## 1. Introduction

Pregnancy increases the risk of thrombosis. APCR phenotype has been associated with venous thromboembolism (VTE), the primary cause of maternal death in developed countries [1–3].

In normal conditions, APC inactivates the coagulant protein active FV(a) by cleaving in an ordered sequence specific sites of FV(a). The first cleavage site is Arginine (Arg) 506, and the second is (Arg) 306 followed by (Arg) 679 [4]. Mutations in the FV gene have been related to APCR. FVL is reported in about 90% of patients with APCR in the general population [5–7]. Other SNPs in the factor V gene which may contribute to inherited APCR either independently or found in association with the FVL mutation include Cambridge Arg^306^, Hong Kong, Arg^306^, the Arg^679^, and the haplotype (H) R2 and R3 polymorphisms. However, reports on the contribution of these mutations to the APCR phenotype are conflicting [7–10].

The pathophysiology underlying APCR not caused by the FVL mutation is still not completely understood. In different studies, it has been suggested that acquired factors might be the cause of APCR in the absence of FV Leiden [11, 12]. A number of coagulation factors can affect the activated partial thromboplastin time (aPTT). Previous literature suggested a possible positive correlation between levels of factors V, VIII and IX and acquired APCR [13]. Protein S and protein C, levels can (or may) affect acquired APCR, but their influence on the resistance seems to be still within the range of normal levels [14]. 

Other known SNPs associated with thrombophilia and adverse outcomes during pregnancy are prothrombin G20210A and MTHFR C677T [15–17]. 

Prothrombin G20210A is associated with an increase in the level of prothrombin protein (FII) in plasma and a resulting 3-fold increase in thrombotic events. The prothrombin G20210A mutation seems to increase the risk of thrombosis in pregnant women by approximately tenfold [18] with the risk of developing obstetric complications increased by fourfold [16].

The MTHFR C677T has been associated with obstetric complications and with birth defects [19, 20]. 

In a previous study in this laboratory, we identified known and novel SNPs in a small number of subjects with APCR determined using the modified Coatest test which did not have the FVL mutation [21].

The main objectives of this study were to (1) determine and compare the levels of factors V, VIII, and IX in the acquired APCR, inherited APCR, and APCR-negative groups, (2) compare the frequency of adverse outcomes in the APCR-positive (acquired and inherited) and APCR-negative groups, and (3) determine the frequency of adverse pregnancy outcomes, associated with thrombophilic mutations other than FVL mutation in our study cohort (*n* = 907). The adverse pregnancy outcomes observed in this study included (previous) recurrent early pregnancy loss (REPL), preeclampsia (PET), and intrauterine growth restriction (IUGR). Pregnancy induced hypertension (PIH), (IUFD) intrauterine fetal dead, and low birth weight (LBW).

## 2. Materials and Methods

### 2.1. Subjects

Ethical approval for the study was obtained from the Research Ethics Committee, and written consent for samples to be collected was obtained from the 907 pregnant women included in this study who attended for routine outpatient gestational screening at the antenatal clinic at University College Hospital, Galway, (UCHG).  Table 1 details the demographics of the study subjects.

Blood samples (Lithium Heparin and EDTA) were collected from subjects between the 16th to 24th weeks of gestation. In this second trimester of pregnancy, little or almost no variation on the coagulation factors has been shown which is appropriate for the assessment of APC status [22]. Testing of APC status before or from 8 to 12 weeks after pregnancy or more frequently during pregnancy would have determined a more accurate stable APC ratio; this is a limitation of the current study. The laboratory analysis of poor anticoagulant response to APC is based on an activated partial thromboplastin time (aPTT) assay. A reduced ratio reflects the reduced rate of inactivation. The cutoff ratio used to determine positive APCR for this study was a value of less than or equal to 2.1 seconds (s). This value was determined by the haematology department and it is in clinical use at UCHG. The cut-off ratio used to determine APCR can vary in sensitivity and specificity depending on the methodology, equipment, and technology used in different laboratories.

### 2.2. APC Status by Classic Coatest Test in Order to Identify Acquired APCR Samples

Blood samples were centrifuged at 4,000 rpm for 5 min, and aliquots of platelet-poor plasma were frozen at −80°C until the assay took place. Plasma was incubated with an equal volume of aPTT reagent for 5 min at 37°C. Clotting was initiated by the addition of CaCl_2_, and clotting times were expressed as a ratio of clotting time in the presence of APC divided by the clotting time in the absence of APC.

### 2.3. APC Status by Modified Coatest Test in Order to Identify Inherited APCR Samples

Blood samples were first diluted 1 in 5 with factor V-deficient plasma that contains a heparin neutralizer, and then it was assayed as described for the APCR classic Coatest test. The addition of the factor V-deficient plasma corrects for deficiencies of other coagulation proteins, neutralises therapeutic concentrations of heparin, and eliminates the effect of some lupus inhibitors [23]. APCR in the presence of factor V-depleted plasma was assessed using the Coatest APCR-kit and factor V-depleted plasma (Chromogenix) [24].

### 2.4. DNA Extraction

DNA was extracted from blood samples for all APCR subjects (*n* = 140) and control samples (negative APCR *n* = 31) using a modification of the CF(12)m-PCR protocol by Ortho-Clinical Diagnostics, Amersham, UK. Spectrophotometric analysis of absorbance at 260 nm was performed in a He*λ*ios *α* spectrophotometer (Unicam Ltd., England) for each DNA sample, and the average yield for DNA was determined to be 30 ng/*μ*L.

### 2.5. Mutation Screening

*PCR-restriction enzyme analysis (PCR-REA)* was applied to identify G20210A in the FII gene, C677T in the MTHFR gene, and FVL, FV Cambridge, FV Hong Kong mutations, and Haplotype (H) R2, allele in the FV gene using modifications of previously published methods [6, 25–29]. DNA probe hybridisation analysis and/or DNA sequencing was applied for Arg^679^ detection as described [21]. One hundred and forty APCR-positive subjects and 31 APCR-negative subjects were tested for all mutations. Our group of control subjects tested for thrombophilic mutations, *n* = 31, was chosen randomly out of the 767 negative APCR subjects identified as normal with the APCR classic Coatest test. A mean of 2.59 and standard deviation of 0.2804 were identified for this negative APCR group of *n* = 767 subjects. Conversely, a mean of 1.745 and standard deviation of 0.1104 were identified for the positive APCR (inherited plus acquired) *n* = 140. The minimum value identified for negative APCR was 2.160 whereas for positive APCR was 1.490. The maximum negative APCR value identified was 3.030, whereas for positive APCR it was 2.060 seconds. Thrombophilic mutations are interfering with the coagulation equilibrium varying the aPTT time related to APC ratio range. 

All mutations were screened in duplicate and positive controls consisting of DNA from heterozygous and homozygous individuals for FVL and MTHFR-C677T mutations, and heterozygous individuals for other mutations investigated were included where it was available. A negative control, consisting of water in place of DNA template, was included in each PCR run. PCR amplification was performed on the GeneAmp PCR System 9600 (Perkin Elmer, USA).

### 2.6. DNA Sequencing Verifying Mutations Identified

DNA sequencing on 50% of the PCR products from subjects screened for the Arg^679^ mutation was performed to verify the normal genotype obtained by Southern blot-DNA probe hybridisation analysis for these subjects. The 400 bp PCR products from Exon 13 containing the Arg^679^ site of the FV gene were purified using the QIAprep Spin Miniprep Kit (Qiagen Ltd, UK) according to the manufacturer's instructions and sent for DNA sequencing to an external sequence service provider (MWG Biotech, Germany). One sample positive for the Cambridge mutation by PCR/REA analysis was also purified using the QIAprep Spin Miniprep Kit (Qiagen Ltd, UK) and sent for sequencing to confirm the Arg^306^ mutation.

### 2.7. Levels of Factors V, VIII, and IX

Levels were determined for the APCR-positive and APCR-negative groups using a one-stage APTT assay on an MDA 180 coagulometer (Organon Teknika, Cambridge, UK), using factors V, VIII and IX deficient plasma's (Diagnostica Stago). The APCR negative group comprised of 50 women selected at random from different batch runs of Coatest tests providing the basis for the control group in comparing data between different variables.

Results for factors V, VIII, and IX levels were expressed as percentage (%) levels, and the reference ranges used [30] are currently in clinical use at the Haematology Laboratory, UCHG. Each run was controlled by the use of a normal (MDA verify 1, Biomerieux) and abnormal control (coagulation control A, Technoclone GmbH).

### 2.8. Statistical Analysis of Pregnancy Adverse Outcomes in the Group Observed in the 907 Women Included in the Study

Data was collected from the Maternity Department at UCHG. The number and type of pregnancy outcomes studied were analysed using SPSS version 17.0 software. The Pearson Chi Square test was used to compare the APCR-positive group and the negative APCR group to analyze the thrombophilic mutations and the range of adverse outcomes associated with each group. The Pearson Chi-Square test was used to compare the number and type of adverse pregnancy outcomes in the inherited versus acquired APCR group. SPSS was used to analyse the levels of coagulation factors V, VIII, and IX using Kruskal-Wallis statistical testing.

### 2.9. Criteria for Adverse Outcomes Diagnosis

PET-BP (Blood pressure) ≥140/90 mmHg, +1 proteinuria and oedema. PIH-BP ≥140/90 with no proteinuria in accordance with the Official Journal of the International Society for the Study of Hypertension in Pregnancy. IUGR is defined as fetal growth of less than 5th percentile for gestational age [25]. LBW is defined as a weight of less than 2500 g (up to and including 2499 g), irrespective of gestational age.

## 3. Results

**Figure 1:**
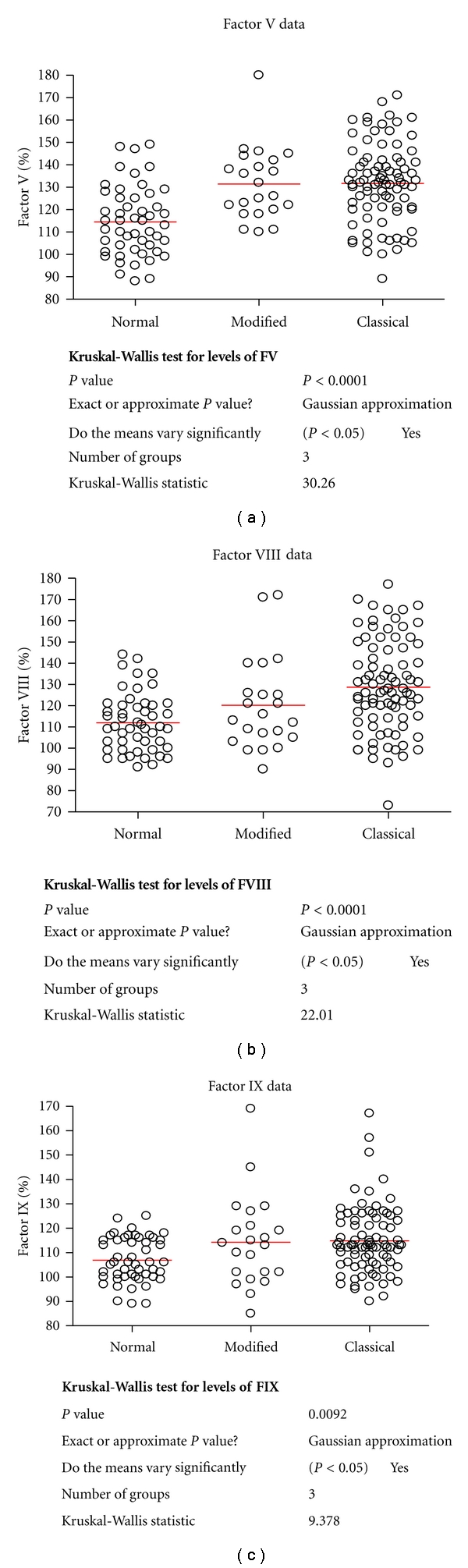
(a) Graph comparing FV levels in differing groups within the study, (b) graph comparing FVIII levels in differing groups within the study, and (c) graph comparing F IX levels in differing groups within the study.

We identified 15.4% (*n* = 140) of the study cohort (*n* = 907) as APCR positive with acquired and/or inherited Coatest tests. Factors V, VIII, and IX levels showed a positive correlation with an acquired APCR phenotype. The levels for each factor in the APCR positive (inherited) and APCR-positive (acquired) and APCR-negative groups are shown in Table 2 and Figure 1. Levels of factors V, VIII, and IX in the acquired APCR group were significantly higher compared to the normal control group. The means were factor V 131.5 IU/dL versus 114.6 IU/dL for the control group, factor VIII 128.7 IU/dL versus 111.9 IU/dL in the control group and factor IX 114.8 IU/dL versus 106.9 IU/dL in the control group. 

Ninety-two subjects (66%) of the positive APCR group had common thrombophilic mutations, 32% of whom had the FVL mutation. A total of *n* = 116 mutations were identified in this group, 68 subjects had one mutation, 23 had two mutations, 1 subject had three mutations, and forty-eight subjects did not have any of the known mutations screened for.

Within the acquired (without FVL) positive APCR (*n* = 105), 70 mutations were distributed among 61 subjects who had one or more of these mutations. Within the negative APCR group (*n* = 31), 18 subjects had one mutation. The distribution of thrombophilic mutations is summarised in Table 3.

Frequencies of adverse outcomes in positive APCR and negative APCR groups were very similar at 35.7% and 34.2%, respectively (Table 4). The comparison of adverse pregnancy outcomes and thrombophilic mutations is summarised in Table 5. PIH showed statistical significant (*P* < 0.05) association with Cambridge and prothrombin G20210A. LBW showed statistical significant (*P* < 0.05) association with HR2 and MTHFR.

## 4. Discussion

The frequency of APCR has been reported as approximately 5% in the general Caucasian population [31, 32]. This varies from 1% to 15% in different countries with a frequency of 3% reported in Italy and Spain and a frequency of 15% reported in Northern Sweden [33]. APCR in the general population and during pregnancy is reported to be most frequently caused by FVL mutation [34–36], inherited APCR. 

Several reports have shown, however, that between 5 and 10% of APCR in Caucasians does not involve the FVL, and the cause of positive APCR in these cases is not known [34, 37–40]. In our study cohort (*n* = 907), the frequency of acquired APCR, determined by the classic Coatest test method, of 11.5%, was substantially higher than the frequency of inherited APCR identified by modified Coatest tests, 3.9%. De Visser et al. demonstrated that an altered APCR in the absence of FVL confers a 2.5-fold increased risk for venous thrombosis [41]. 

In this study, a significant statistical correlation of the acquired APCR phenotype with Factors V, VIII, and IX levels was demonstrated. Further study would be required to determine the interaction of coagulation factors to further characterize this acquired APCR phenotype in early pregnancy. Acquired APCR can be the result of a complex series of reactions giving a defective APC-mediated degradation of these coagulation factors. In turn, an increase of these coagulation factors in early pregnancy stages could cause an imbalance in the coagulation cascade and lead to vascular and endothelial damage during implantation and placentation. This has the potential to cause placental infarcts and high levels of fibrin deposition, which has been previously associated with APCR [42]. Spontaneous abortion may take place in response to an increased coagulation imbalance, and if there are other thrombotic factors including thrombophilic mutations interacting simultaneously when the subject is beyond the 20th week of pregnancy, the damage could develop into PET or PIH.

Mutations in the FV gene excluding FVL, particularly at the other cleavage sites of FV, Arg^306^, and Arg^679^, have the potential to contribute to the APCR phenotype [6, 43]. Haplotype (H) R2 has also been associated with mild APCR when found in combination with the FVL [7, 10, 44, 45]. There have been a number of other mutations identified in the FV gen; however, a larger pregnant population would need to be studied to determine if these mutations contribute significantly to APCR in the Caucasian populations [21, 46–48]. Thrombophilia during pregnancy has been linked with mutations other than those located in the FV gene including prothrombin G20210A and MTHFR-C677T [17, 49, 50]. In this study, we identified thrombophilic mutations in 66% of our positive APCR (acquired plus inherited) group (*n* = 140). One of those subjects had 2 mutations in the FV gene, FVL and FV Cambridge-G1091C, at two cleavages sites of FV for APC [21]. This subject was also found in this study to be a carrier of MTHFR-C677T mutation. Previous studies established that the presence of more than one prothrombotic polymorphism is associated with a substantial risk of VTE with a high risk of adverse pregnancy outcomes [51, 52]. In addition, more recently MTHFR-C677T mutation has been implicated in pre-eclampsia. The high frequency of MTHFR-C677T in the negative APCR and positive APCR groups may underlie populations-specific differences [53]. Discrepancies in the adverse pregnancy outcomes associated with MTHFR-C677T may be explained by the specific frequency differences among populations [54, 55].

We also identified prothrombin G20210A in 3 subjects in the acquired APCR group without the presence of FVL mutation, with the (H) HR2 haplotype and MTHFR-C677T identified in both positive APCR (acquired plus inherited) and negative APCR groups. 

The negative APCR samples (*n* = 31) tested for thrombophilic mutations in this study to represent the normal cohort are a small sample number, and this may be a flaw in our study resulting in nonsignificant differences. Testing a larger number of negative APCR would have given more confident results. This can be something to be investigated in a future study. 

While some previous studies have shown an association of positive APCR with PET, IUGR, and IUFD [55] in this study there was no significant difference in the frequency of adverse outcomes between positive APCR and negative APCR groups. This seems to be consistent with other previous studies [56]. Nonetheless, the overall frequency adverse pregnancy outcomes in the positive APCR makes this study group “a high-risk group.” Environmental factors such as smoking and body mass index (BMI) that could have influenced the rate of acquired APCR and the frequency of adverse outcomes identified, were not included. This is a limitation of the study. 

In our total study group, thrombophilic mutations including FVL Cambridge and Prothrombin G20210A seem to be related to PIH, whereas MTHFR-C677T and HR2 haplotype appeared to be associated with LBW. Although positive APCR by itself does not seem to be the cause of severe adverse outcomes during pregnancy, inherited APCR can nevertheless have an effect on hypertension when it is present in combination with other known and unknown thrombophilic risk factors acting simultaneously during pregnancy.

## Figures and Tables

**Table 1:** Demographics of study cohort (*n* = 907) pregnant woment attending for antenatal care at UCH, Galway.

	Positive APCR		Negative APCR	
Distribution of parity by total population				

Parity	Freq	%	Freq	%

Primigravida	59	42.2	301	40.3
Multigravida	81	57.8	226	59.7
Data missing	0	0	15	1.9
Total	140	100	767	100

Distribution of maternal age by total population				

Maternal age	Freq	%	Freq	%

<19	12	8.6	38	5.1
20–29	29	20.7	143	19
30–39	87	62.1	473	62.9
40 < 45	12	8.6	98	13
Data missing	0	0	15	0
Total	140	100	767	100
range	15–42		16–45	
mean	28		29	

Distribution of mode of delivery by total population				

Mode of delivery	Freq	%	Freq	%

Spontaneous vaginal delivery	82	58.6	456	60.6
Assisted vaginal	27	19.3	121	16.1
Cesarean Section	29	20.7	158	21
Data missing	2	1.4	32	2.3
Total	140	100	767	100

Distribution of mode of delivery by total population				

Mode of delivery	Freq	%	Freq	%

SVD	82	58.6	456	60.6
AV	27	19.3	121	16.1
CS	29	20.7	158	21
Data missing	2	1.4	32	2.3
Total	140	100	767	100

SVD: Spontaneous vaginal delivery; AV: Assisted vaginal; CS: Cesarean section.

**Table 2:** Comparison of factor V, VII and IX in positive APCR acquired and inherited and negative APCR groups.

	Positive APCR		Negative APCR
Correlation between levels of the coagulation factor V and APCR			

Factor V levels	Acquired APCR	Inherited APCR	

Number of values	85	22	50
Minimum value	89	110	88
Maximum value	171	180	149
Mean	131.5	131.7	114.6
Coefficient of variation	13.35%	12.34%	13.49%

Correlation between levels of the coagulation factor VIII and APCR			

Factor VIII levels	Acquired APCR	Inherited APCR	

Maximum value	177	172	144
Minimum value	73	90	91
Mean	128.7	120.2	111.9
Coefficient of variation	16.79%	18.10%	12.14%

Correlation between levels of the coagulation factor IX and APCR			

Factor IX levels	Acquired APCR	Inherited APCR	

Number of values	85	22	50
Minimum value	90	85	89
Maximum value	167	169	125
Mean	114.8	114.2	106.9
Coefficient of variation	11.93%	16.25%	8.53%

**Table 3:** Distribution of the thrombophilic mutations identified in our study cohort.

APC	Mutations identified/subjects tested	Frequency from the subset of subjects tested in each group (*1) (*2), (*3).	Frequency from the total cohort *n* = 907	Thrombophilic mutations identified
(*1) *n* = 140	Positive APCR (inherited and acquired)			

	67/140	47.8%	7.3%	MTHFR-C677T (54ht13hom)
	3 /140	2.1%	0.3%	ht prothrombin G20210A
	16/140	11.4%	1.7%	ht (H) R2
	29/140	20.7%	3.1%	ht FV Leiden
	1/140	0.7%	0.1%	ht Cambridge
	48/140	31.4%	3.9%	no mutation

(*2) *n* = 105	Positive APCR (acquired)			

	51/105	48.5%	5.6%	MTHFR -C677T (38ht13hom)
	3/105	2.8%	0.3%	ht Prothrombin G20210A
	16/105	15.2%	1.7%	ht(H) R2
	44/105	41.9%	4.8%	no mutations

(*3) *n* = 31	Negative APCR			

	5/31	16.1%	0.6%	ht (H) R2
	13/31	41.9%	1.7%	MTHFR-C677T
	13/31	41.9%	1.7%	No mutation

(*1) *n* = 140 are subjects with total APCR (acquired +modified).

(*2) *n* = 105 are subjects with acquired APCR, without inherited FVL.

(*3) *n* = 31 are negative APCR subjects tested out of 767.

*n*: Number of subjects; ht: Heterozygotes; hom: Homozygotes; H: Haplotype.

Subjects identified with more than one mutation simultaneously:

in the total APCR group, (inherited plus acquired) They had 13 subjects with MTHFR+FVL; 1subject with MTHFR + FVL + Cambridge; 7 subjects with MTHFR + HR2; 1 subject with prothrombin G20210A + HR2; and 1 subject with prothrombin G20210A + MTHFR; in the acquired APCR group, They had 7 subjects with MTHFR+HR2; 1 subject with prothrombin G20210A + HR2; 1 subject with prothrombin G20210A + MTHFR.

**Table 4:** Frequency of adverse outcomes identified in positive APCR (acquired plus inherited) versus negative APCR. *P* value from Pearson Chi-Square test applied to positive APCR (acquired plus inherited) versus negative APCR.

		Total outcomes frequency	EPL	PET	PIH	IUGR	IUFD	LBW
Positive	*N* = 140	*N* = 50	*N* = 38	*N* = 3	*N* = 12	*N* = 1	*N* = 0	*N* = 37
APCR	Freq.%	35.7%	27.1 %	2.2%	8.6%	0.7%	0%	26.8%

Negative	*N* = 767	*N* = 262	*N* = 196	*N* = 39	*N* = 37	*N* = 20	*N* = 3	*N* = 256
APCR	Freq.%	34.2%	25.7%	5.2%	4.9%	2.6%	0.4%	34.5%

Pearson Chi-Square	*P* value	0.722	0.712	0.125	0.077	0.168	0.456	0.080

**Table 5:** Results from Pearson Chi-Square test. *P* values for the association between each adverse pregnancy outcome identified in this study and thrombophilic mutations indentified in our total study cohort *n* = 907.

*P* values	Presence of outcomes	EPL	PET	PIH	IUGR	IUFD	LBW
FVL	0.237	0.832	0.748	**0.004**	0.396	0.751	0.289
Cambridge	0.469	0.554	0.822	**0.000**	0.877	0.954	0.480
HR2	0.951	0.654	0.194	0.466	N/A	N/A	**0.016**
Prothrombin	0.426	0.133	0.928	**0.008**	0.413	0.883	0.907
MTHFR	0.859	0.203	0.428	0.584	N/A	N/A	**0.048**

NA: no statistics are computed because there is no IUGR in the MTHFR and HR2 groups.
